# Analysis of the consultations on the InfoChagas digital platform

**DOI:** 10.1371/journal.pntd.0013469

**Published:** 2026-01-22

**Authors:** Elizabeth Posada Diago, Inés María Iglesias Rodríguez, Irene Losada-Galván, Edelweiss Aldasoro Irastorza, Pau Rubio Figuerola, Cristina Alonso-Vega, Joaquim Gascon, Mirko Rojas-Cortez, Rafael Herazo, Andrea Marchiol, Marcelo Abril, Elvira Hernández, Julio Alonso-Padilla, Francisco Javier Sancho Mas

**Affiliations:** 1 ISGlobal, Barcelona, Spain; 2 School of Tropical Medicine and Global Health, Nagasaki University, Nagasaki, Japan; 3 CIBER de Enfermedades Infecciosas, Instituto de Salud Carlos III (CIBERINFEC, ISCIII), Madrid, Spain; 4 Facultat de Medicina i Ciències de la Salut, Universitat de Barcelona (UB), Barcelona, Spain; 5 The International Foundation for Integrated Care (IFIC), Schiphol, The Netherlands; 6 Fundación SANIT (Salud Naturaleza Integral), Cochabamba, Bolivia; 7 Drugs for Neglected Diseases initiative (DNDi), Rio de Janeiro, Brazil; 8 Independent consultant, Buenos Aires, Argentina; 9 FINDECHAGAS (Federación Internacional de Asociaciones de Personas Afectadas de Chagas), Xalapa, Mexico; 10 AMEPACH (Asociación Mexica de Personas Afectadas por Chagas), Xalapa, Mexico; 11 Chagas Disease Global Coalition, Barcelona, Spain; Huazhong University of Science and Technology Tongji Medical College, CHINA

## Abstract

**Background:**

People affected by *T cruzi* infection, and at risk to develop Chagas disease, face significant barriers to accessing healthcare and often lack adequate information and communication resources. To address this, the Chagas Coalition developed the InfoChagas digital platform, including an informative website and consultations sections, linked YouTube channels in English and Spanish.

**Methodology:**

This cross-sectional study analyzed consultations to the InfoChagas platform between 2016 and 2023. We conducted quantitative and qualitative analyses of the consultants’ profiles, enquiry topics, and barriers that people at risk of having *T. cruzi* infection faced when seeking health care.

**Principal findings:**

A total of 272 consultations were received via the website (n = 129) and YouTube (n = 143) from 19 endemic and non-endemic countries. Nearly two-thirds of these were from Argentina (n = 33, 25.6%), Mexico (n = 24, 18.6%), and Bolivia (n = 22, 17%). Main topics included doubts related to access to treatment (n = 71, 26%), transmission risk in daily life (n = 38, 14%) and requests for help or referrals to health care professionals (n = 38, 14%). Half of the users (n = 138, 50.7%) reported barriers to healthcare access, with the majority citing the inability of the system to provide adequate and effective care for *T. cruzi* infection (n = 127, 92.7%). Additionally, 96.7% (n = 270) of the narratives highlighted the need for information, education, and communication (IEC) to understand the complexity of *T. cruzi* infection and to demystify concepts that hinder access to healthcare.

**Conclusions/significance:**

InfoChagas serves as an informative, communicative, educational, and supportive resource for individuals affected by or at risk of *T. cruzi* infection across diverse geographic regions. Given the critical role of IEC strategies and the rise of healthcare technology, digital tools such as InfoChagas is a useful resource to face the existing barriers to healthcare access for the infection and to improve access to quality information and healthcare services.

## Author summary

*Trypanosoma cruzi* infection remains a significant global health challenge, with many individuals at risk facing difficulties accessing essential information and healthcare. To address these barriers, the InfoChagas digital platform was created, offering free information and professional consultations. Our analysis of consultations from 2016 to 2023 revealed that most inquiries came from high-burden countries, with users primarily seeking information on treatment access, transmission risks, and referrals to healthcare services. Many expressed challenges in obtaining proper care, emphasizing the need for improved information and communication strategies. InfoChagas has proven valuable in translating scientific knowledge into practical, accessible resources, especially for populations with limited healthcare access. Our findings also highlight the need to improve digital health literacy and develop comprehensive educational strategies to empower those affected by *T. cruzi* infection. This study underscores the potential of digital platforms to complement healthcare systems, improve access to critical health information, and contribute to global efforts to control and manage *T. cruzi* infection.

## Introduction

Chagas disease is a neglected tropical disease (NTD) caused by the protozoan parasite *Trypanosoma cruzi* (*T. cruzi*) [1]. It affects more than 6 million people worldwide, exerting its greatest burden in the Americas, where the infection is endemic in 21 countries [1]. However, due social, cultural, political, economic and clinical barriers, including the silent course of the infection and the limited accessibility and sensitivity of conventional serological tests, it is estimated that 99% of those infected do not have access to diagnosis and treatment [1]. Furthermore, only a few countries have policies that cover the control of several forms of transmission as well as the existence of a comprehensive care system for people with *T. cruzi* infection, all of which largely prevent their access to healthcare [1–4]. In general, the population affected by *T. cruzi* infection and those at risk have limited opportunities for information and communication or educational resources for their questions, doubts or concerns related to *T. cruzi* infection [5,6]. Therefore, in recent years the information, education and communication (IEC) in *T. cruzi* infection has gained great importance in some context, and has been recognized as an essential strategy to control the impact of the infection, as the socio-cultural dimension plays an important role in the healthcare seeking behavior of those at risk [5,7,8].

Alongside traditional IEC approaches, digital tools have increasingly supported community engagement and access to information. For example, mobile and web-based platforms for vector identification and citizen-science initiatives, such GeoVin, TraeTuChipo, or Triatokey, have proven effective for entomological surveillance, but generally do not provide individualized guidance for people affected by *T. cruzi* infection [9–11]. Telemedicine interventions, including pilot teleconsultations for *T. cruzi* infection and other NTDs, have shown that remote, user-initiated consultations are feasible and acceptable, yet these services are typically limited to specific hospitals or national programs and lack broad, geographically accessibe coverage [12].

To respond to this need for information and support, in a context of increasing use of technology and digitalization for health communication for patient care, the InfoChagas digital platform was created as part of the Chagas Coalition in 2014. It was conceived as a website, infochagas.org, (in Spanish and English) that includes an information and advice section, as well as informative links to videos and slideshows that advocate and educate about different aspects of *T. cruzi* infection [13–16].

**Fig 1:**
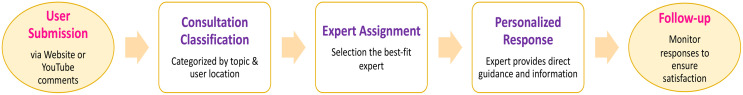
Workflow of the InfoChagas reporting and consultation management process.

The advice section of the website offers people, directly or indirectly affected by *T. cruzi* infection worldwide, the opportunity to consult their questions and doubts and receive personalized information and advice from an expert. Depending on the topic of the consultation and/or the location of the InfoChagas user, the Coalition selects from its network of experts those who has more expertise on the subject in order to address it with more efficiently. A Coalition collaborator then follows up the process to ensure that the consultation is resolved and that the user is satisfied. In addition, the YouTube channels offer a visual and easy-to-understand format in which InfoChagas collaborators respond to comments on the YouTube channel following the same process than in the website, offering guidance and updated information on the issues raised [17]. Fig 1 presents the key stages of the reporting process within the InfoChagas platform and the subsequent management of user requests. This study aims to analyze the questions and narratives received on the InfoChagas platform between January 2016 and December 2023 to identify users’ informational needs, concerns and barriers related to *T. cruzi* infection. By characterizing these community-expressed needs, the study seeks to generate evidence that can guide improved health communication, strengthen support mechanisms, and inform strategies to enhance access to diagnosis and care for people affected by or at risk of *T. cruzi* infection.

## Materials and methods

### Ethics statement

The study was approved by the Ethics Committee of the Hospital Clinic of Barcelona (ref.: HCB/2023/0138). We analyzed the profile of the users, the topics of the consultations, and the main barriers expressed by the consultants during their healthcare seeking process.

### Study design

The study followed a cross-sectional design with the aim of retrospectively analyzing consultations received on the InfoChagas platform, including the website and YouTube channels, between January 2016 and December 2023.

### Data collection

We define a consultation about *T. cruzi* infection as the action or process of seeking information, guidance or recommendation related to any of the aspects of the condition including prevention, transmission, diagnosis, treatment, management of complications, public health, socio-cultural and psychosocial, in order to get an advice or opinion. Therefore, in the YouTube channels, the illegible and non-consultation-oriented comments, such as personal opinions or non- *T. cruzi* infection related comments, were excluded. All remaining comments were manually screened by two researchers to ensure they met the study definition of a consultation. After filtering for the *T. cruzi* infection -related consultations in the YouTube channels, in English (YouTube-EN) and in Spanish (YouTube-ES), a total of 143 consultations received between January 2016 and December 2023 were included in the analysis. In addition, all consultations received on the website (n = 129) from April 2019 until December 2022 were also included. Consultations received from the website before 2019 were not included due to the difficulties in accessing the data.

The variables collected from the YouTube channels and the InfoChagas website included the content of the consultation, the year it was made, and whether it was made by a person who self-identified as affected by *T. cruzi* infection or not. However, the socio-demographic characteristics, such as gender and country of origin, were only collected for the questions received through the website, designated as required fields in the consultation forms to ensure systematic and complete capture of this information.

### Data analysis

The data were analyzed quantitatively and qualitatively to increase the robustness of the findings. Quantitative descriptive analysis provided an overview of user profile, the themes of the consultations, and the barriers reported were analyzed descriptively, expressed as frequency counts and percentages. On the other hand, the narratives analyzed qualitatively through thematic analysis using an inductive reasoning approach and organizing with the support of Microsoft Excel, offered deeper insights into the challenges and informational needs expressed in the consultations.

Before initiating the formal thematic analysis, both researchers independently coded a small subset of consultations to align interpretations and ensure consistency in the subsequent coding process. The thematic analysis was structured in six steps: familiarization with the data, codification, development of the themes, review, labelling and writing [18]. Quotations that were more representative for the analysis were translated into English for publication.

The thematic analysis of consultations and barriers was coded independently by two researchers with different backgrounds, an anthropologist and a physician, both with expertise in *T. cruzi* infection. If the result of the coding differed or if they encountered concepts outside the coding scheme, they discussed adjustments until reaching a consensus to ensure consistency and reliability of the final coded dataset. The researchers were fluent in Spanish, Portuguese, and English.

In order to analyze the barriers expressed by the users, we considered the definition of barriers proposed by Forsyth et al., as: “…any factor that either limits the availability of, or prevents patients from accessing diagnosis, treatment, and/or clinical management of *T. cruzi* infection “ [19]. Therefore, we used that framework to classify the barriers into four main categories: (i) systemic (limitations of the health care system to provide effective and appropriate care for people with *T. cruzi* infection); (ii) structural (inequalities within the political-economic system that limit access to health care for marginalized groups); (iii) clinical and pathophysiological (related to the biological characteristics of *T*. *cruzi* that challenge the access and efficacy of testing and treating); and (iv) psychological [19]. Primary barriers were identified directly from the consultations, while secondary barriers and their concurrences were examined through in-depth qualitative coding using ATLAS.ti. Co-occurrence matrices were generated to identify overlaps between barrier categories and consultation types. Because co-occurrence is calculated only when multiple codes appear within the same narrative segment, consultations containing a single primary barrier do not contribute to co-occurrence counts. Regarding to systemic barriers, due to their large number, led us to identify recurrent patterns in the consultation narratives, which we consistently coded into four subcategories to enable more detailed analysis: lack of information about the process of seeking care; limited access to diagnostic and antiparasitic treatment; lack of information about the follow-up of chronic cases; and limited knowledge of the infection among health care providers.

## Results

### User characteristics

**Fig 2:**
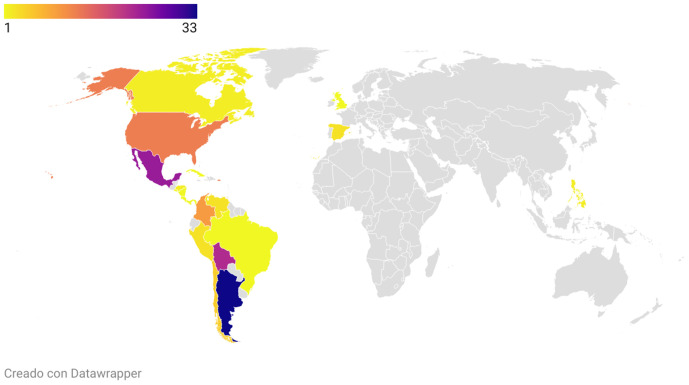
Consultations by country. Chart created with Datawrapper (Datawrapper. 2025: https://app.datawrapper.de/).

A total of 272 consultations were received, after excluding non-consultations-oriented comments: 129 were from the website, 135 from the YouTube-ES, and 8 from the YouTube-EN. On the website, 44% (n = 57) of the users were men and 56% (n = 72) were women. Although the users came from 19 different countries and three different continents (the Americas, Europe, and Asia), almost two-thirds of the website consultations came from Argentina (n = 33, 25.6%), Mexico (n = 24, 18.6%), and Bolivia (n = 22, 17%) (Fig 2).

**Fig 3:**
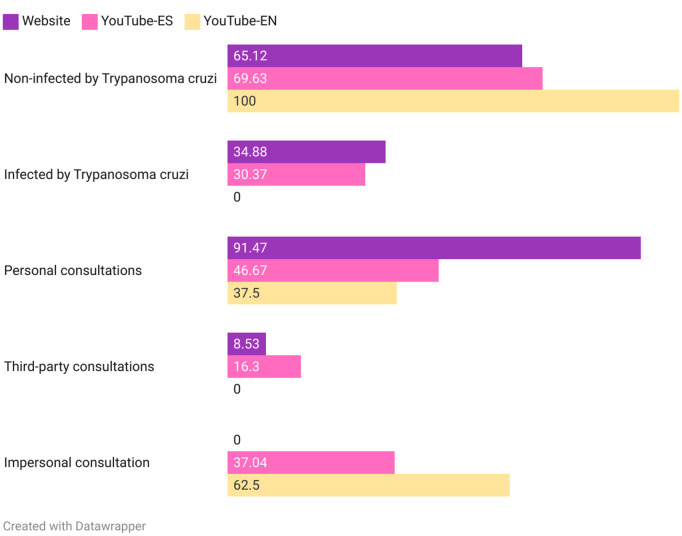
Characteristics of the users (shown as percentage). Plot created with Datawrapper 2025.

As shown in Fig 3, more than half of the users did not identify themselves as having or not having the infection with *T. cruzi* (n = 186, 68.4%). Although most of the consultations on the website sought answers for the users themselves (n = 118, 91.5%), this tendency did not represent the majority of the questions on the YouTube channels, where general inquiries unrelated to the consulter’s personal health, referred to as impersonal questions, were balanced with the personal ones.

### Activity of the InfoChagas tool

**Fig 4:**
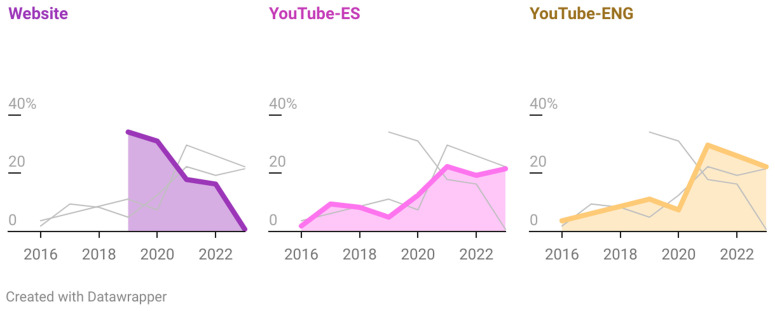
Activity of InfoChagas between January 2016 to December 2023 (shown as percentages). Graph created with Datawrapper 2025.

On the website, almost two-thirds of the consultations, 65% (n = 84), were from 2019 and 2020. In contrast, on the YouTube channels, most of the activity (77.8% for YouTube-EN and 63% for YouTube-ES) was received from 2021 to 2023 (Fig 4).

During the period of the study, between March 2019 and December 2022, the InfoChagas website recorded 619,045 unique users, 2,645,579-page views, and 740,883 sessions, with an average session's duration of 1 minute and 42 seconds. Approximately 10.9% of users were returning visitors. The majority of site traffic was derived from mobile devices (65.63%), followed by desktop computers (32.98%) and tablets (1.39%).

In terms of geographic distribution, the largest proportion of users originated from Argentina (26.92%), Mexico (13.77%), and Bolivia (13.00%), while users from other endemic regions in Latin America accounted for 39.34%. A smaller group of users (0.57%) came from non-endemic Latin American countries, predominantly the Dominican Republic and Puerto Rico. Additionally, 10.11% of users were located in non-endemic regions outside Latin America, including North America, Europe, Asia, and Oceania. A small proportion (2.07%) of users were categorized under the “not set” category.

The primary source of traffic to the site was organic search, contributing to 87.6% of total visits, followed by direct traffic (12.5%) and minor contributions from referral (0.6%) and social media (0.2%) sources.

Regarding content engagement, data was available for 86.7% of sessions (642,411). The most frequently visited topics were related to the transmission and symptoms of *T.cruzi* infection, with “Life Cycle of the Parasite” (17.61%), “How It Is Transmitted” (17.02%), and “Symptoms of Chagas” (13.22%) being the most accessed. Other prominent topics included “How Is It Diagnosed” (11.33%) and “Treatment” (6.81%). The section “Expert Consultation” received the lowest number of visits, with only 47 sessions recorded (0.01%).

The YouTube platform itself is divided into eight videos on the cycle of the parasite, general information on *T. cruzi* infection, symptoms, diagnosis, transmission, treatment, prevention, and research. Considering that all these videos were published in 2014, the most popular video in terms of visualization, likes and comments was the one related to the cycle of the parasite*,* with 687,000 views in the YouTube-ES and 91,535 views in the YouTube-EN by December 2023. The least popular video was the one on *T. cruzi* infection research (530 views on YouTube-ES and 158 views on YouTube-EN).

### Characteristics of the consultations

After analyzing the themes of the consultations, the following eleven themes emerged: (1) prevention or screening, (2) vector control, (3) public health, (4) mother-to-child transmission, (5) other modes of transmission, (6) symptoms or clinical course, (7) diagnosis, (8) treatment, (9) requests for help or referral, (10) socio-cultural, and (11) requests for other type of information linked to *T. cruzi* infection.

We observed that 96.7% (n = 270) of the consultations (n = 272) analyzed expressed a need for information and education to people at risk of having the infection and relatives, especially features that provide the person at risk of *T. cruzi* infection with the necessary insight to understand the complexity of *T. cruzi* infection in order to acquire an active role in the health care decision-making process.

**Fig 5:**
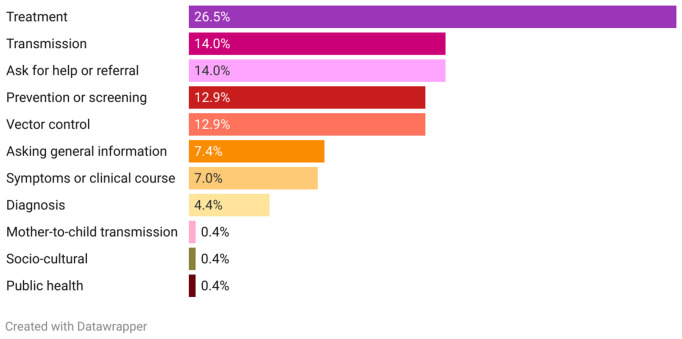
Topics of consultations (shown as percentage). Plotted with Datawrapper 2025.

Quantitatively analyzing the characteristics of the consultations (n = 272), the most recurrent topic was access to treatment (n = 71, 26%), followed by doubts about the transmission of the infection (n = 38, 14%) and asking for help or referral in their area (n = 38, 14%). Vector control and doubts about how to be screened or diagnosed were also among the most frequently mentioned topics (Fig 5).

The treatment consultations analyzed reflected a need for a deeper understanding of the role of anti-parasitic and symptomatic treatments for the infection, where to access treatment, and how to self-manage adverse events during treatment. Below are some representative examples of the consultations received (translated into English):

- *“I wanted to know what treatments or cures are there? I have Chagas disease. The electrocardiogram shows that I have arrhythmia, right bundle branch block.” (Woman from Buenos Aires, Argentina).*
- *“My father has Chagas disease. He is 50 years old and got it when he was 26. Can he still be cured?” (Man from Santa Cruz, Bolivia).*
- *“Hello, today is my 27*^*th*^ *day out of 60. I feel very nervous, I have no appetite. I feel like my throat is tight. I have not slept for two days. What do you recommend I do? Please help”. (Woman from Jalisco, Mexico).*

In terms of transmission, there is a need to translate scientific knowledge into real-life situations, reflected in daily life scenarios, family situations, or perceptions of the risk in the work environment. Some of the consultations, translated into English, are shown below:

- *“Hello, I would like to know if Chagas disease can be transmitted through menstrual blood during sexual relations”. (Woman from Guanajuato, Mexico).*
- *“Hello. I am a nurse and I had an accident at work while inserting a nasogastric tube in a patient with Chagas disease. Gastric fluid splashed into my eyes and mouth. I wanted to know if I am at risk of infection”. (Woman from Neuquen, Argentina).*
- *“Good evening. I work in a children’s home and we about to admit a one-month-old baby admitted who has been diagnosed with Chagas. Are the workers at risk of infection?” (Woman from Buenos Aires, Argentina).*

Asking for help or referral is the third most repeated request, and is more present in the YouTube-ES.

- *“Hello, my friend has been diagnosed with Chagas disease. What can I do? Please help me. I am from Bolivia, Riberalta”. (User from Riberalta, Bolivia).*
- *“I have Chagas disease. Where can I find the program in La Paz?” (Woman from La Paz, Bolivia).*
- *“Doctor, I need your help, please. My sister has this problem and it is very serious. I beg you to help us…”. (User from an undisclosed location).*

Consultations about the presence of the vector in or around the household, and asking for guidance on what to do next steps were received from several countries, including those considered non-endemic, where triatomines are not present.

- *I wanted to know what the procedure is if we suspect that we have killed a vinchuca? Whether at home or somewhere else. We must notify the health authorities? (Woman from Buenos Aires, Argentina).*
- *“I’m like totally freaking out right now. I thought these bugs were native to Texas. I bet I can collect a half dozen this afternoon? Or perhaps I’m mistaking its twin?” (user from the United States of America).*
- *“Greetings! We are a family in the Philippines long plagued by kissing bug bites. Is there a strong possibility that we are also infected with Chagas?” (Man, Metro Manila, the Philippines).*

The lack of information on when, where, and how to be tested is generalized in the different countries and coming from users with different backgrounds, including healthcare professionals.

- *“Good morning, I am a Peruvian doctor and I am interested in taking the Chagas test - in Spain - (based on my medical history and symptoms). I would appreciate it if you could inform me of the most direct and quickest way to access the test”. (Man from Barcelona, Spain).*
- *“After being bitten, how long should I wait to have a test done?” (Man from Chiapas, Mexico).*
- *“Doctor, what is the name of the test to see if a person has Chagas disease, please?” (user from an undisclosed location).*

### Answering consultations

All consultations on the website and more than half of those on the YouTube channels were answered by a health professional from the InfoChagas team. In the case of YouTube, almost 10% (9.6%; n = 13) of the consultations not answered by the InfoChagas team were correctly answered online by people outside the InfoChagas team. However, a third of the consultations on YouTube remained answered (25.1%; n = 35) or received poor online answers from people outside of the team (5.6%; n = 8). Five percent (5%; n = 7) of the consultations were conversations between people at risk or infected sharing their experiences or advice.

### Barriers

Following the classification framework described in Methods, barriers were grouped into four categories: systemic (related to health system capacity to provide effective care), structural (inequalities limiting access for marginalized populations), clinical (biological or pathophysiological challenges), and psychological.

One hundred and thirty-eight consultations (n = 138, 50.7%) expressed as the primary barriers to accessing the health system. Although we found barriers related to current clinical challenges associated with the physiopathology of the *T*. *cruzi* infection, to inequalities of vulnerable populations, or psychological barriers, the vast majority of the barriers were related to the capacity of the health system to provide adequate and effective care, systemic barriers (n = 127, 92.7%).

**Fig 6:**
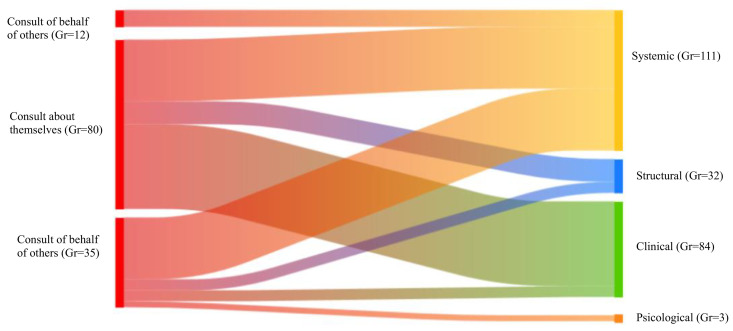
Sankey diagram illustrating the co-occurrence of barrier categories with consultation types in InfoChagas consultations. Created with Atlas-ti. Gr: Groundedness: the number of times a code appears within the dataset.

Narrative analysis revealed that consultations often contained multiple secondary barriers, totaling 230 barrier instances across 272 consultations (84.6%). The Fig 6 presents a Sankey diagram illustrating the qualitative analysis of narrative co-occurrences between consultation type and reported barriers. Due to the characteristics of co-occurrence analysis, the number of actor–barrier links visualized in the Sankey diagram corresponds to coded co-occurrences rather than to the total number of consultations reporting barriers. Structural barriers were reported across all consultation types, self-directed, impersonal, or on behalf of others. Clinical barriers were predominantly reported in self-directed consultations, whereas psychological barriers appeared exclusively in impersonal consultations.

The narratives reflect a perpetuation of classic *T. cruzi* infection myths, such as the lack of any possible health care, a strict age limit to offer the anti-parasitic treatment, the association of *T. cruzi* infection with death, and the preference of “not doing” to avoid “awakening” the parasite, which are the most repeated along the consultations:

- *“...excuse me, I have a question. Could you tell me how to start my treatment for Chagas? I am 36 years old... the doctor told me that there is no cure and that nothing can be done. We can only monitor each year until we reach the grave, literally. That is why I am coming to you, please. Thank you in advance”. (user from an undisclosed location).*

### Systemic barriers

Almost half of the systemic barriers (n = 52, 41%) reflected a lack of health education and public awareness initiatives to guide the health seeking process when the person may be at risk of *T. cruzi* infection. A representative example was:

- *“I found a kissing bug on my back porch by my door, and confirmed it was one with my exterminator. What steps should I take next?” (Woman from Arizona, United States of America).*

The second most common barrier is related to access to the diagnosis and antiparasitic treatment, accounting for 37% (n = 48), as it was reflected in the analysis of the consultations:

- *“I live in El Salvador, I suffer from Chagas here, there is no medicine, I have not been able to find it, I would like to know if I can buy it in another country”. (User from El Salvador).*

The lack of information from the health institutions about the follow-up of patients with chronic infection and the lack of knowledge among health care providers are reflected in the remaining barriers (n = 28, 21.5%), as it is shown in the following note:

*Hello, we recently confirmed 13 kissing bugs in our home, and my husband was bitten multiple times. The doctor will not test unless he has symptoms. Is this accurate? Thank you! (Woman from Arizona, United States of America).*

### Structural, clinical and psychological barriers

Although only 7.3% of the identified barriers were related to structural, clinical, and psychological factors, these barriers were still present. Examples are provided below.

- Structural barrier: *“I lived in Guatemala for 17 years, tried to donate blood there, and was told I had Chagas. Nobody in the U.S. will test me because of insurance. How do I get retested?” (Woman from Washington, United States of America)*
- Clinical barrier: *“I wanted to ask a question: I completed a treatment with benznidazole, but after doing a new test, it still came out positive. Can I do the treatment again?” (Man from Santa Cruz, Bolivia)*
- Psychological barrier: *“How bad, I went to donate blood and got this surprise. I’m scared”. (user from an undisclosed location).*

## Discussion

To the best of our knowledge, InfoChagas was the first virtual platform to provide support, information and education to people affected by or at risk of acquiring *T. cruzi* infection, using a broad geographical approach. Today, there are several websites that provide counselling and information resources at local or national level, but none of them provides individualized consultations for the people affected either in endemic or in non-endemic countries worldwide [9,20–23].

### Geographical reach and user demographics

Although *T. cruzi* infection is a global problem, most of the burden is in Latin America. As a result, InfoChagas received the majority of consultations from Argentina, Mexico and Bolivia. Bolivia has the highest prevalence of *T. cruzi* infection worldwide, while Argentina and Mexico are the second and third countries with the highest number of people with the infection, accounting for 22.3% and 12.9% of the total respectively [24]. Although Brazil has the highest absolute number of *T. cruzi* infection cases with almost two million people affected (26.3% of the total), the very low number of consultations originating from Brazilians could be due to the absence of Portuguese as a language in InfoChagas [24].

Consultations from non-endemic countries such as the United States of America, Canada and Spain were anticipated, given the significant number of migrants from Latin America [24]. The classification of the United States as non-endemic could be debated, given the ongoing presence of transmission cycles of the parasite, as evidenced by consultations regarding the presence of the vector in the country [25,26]. Unexpectedly, questions about vector transmission also came from the Philippines, a non-endemic country that nonetheless has triatomine bugs, as well as from other Asian countries [27–29]. However, the transmission risk information remains limited although *Triatoma rubrofasciata* is present in different countries of Asia [27–29].

Another distinguishing feature of access to website consultations was the gender balance, with only a slightly higher representation of women. This contrasts with the strong feminization of face-to-face consultations for *T. cruzi* infection [30,31]. The low access of men to the health care system has been explained by their reluctance to seek help until severe symptoms appear, in order to preserve the traditional masculine role [32]. However, the higher participation of men in our digital platform could be explained by the ease of access to a space with less social exposure. Although the screening of women is one of the most important strategies to control *T. cruzi* infection due to their important role in mother-to-child transmission, it is important to understand men’s seeking behaviour, considering that male sex has been identified as a risk factor for progression to chronic Chagas cardiomyopathy, with an observed higher risk of morbidity [24,33].

### Reach and interaction

In addition to the wide demographical coverage of InfoChagas, we observed that served as an alternative source of information, that complements conventional health services easily accessible via smartphones. This not only democratizes access to health information in countries where most of the services are out-of-pocket or have low coverage in remote areas, but it is also a convenient resource in the contemporary societal demands or in situations where access to healthcare is limited. Furthermore, as a free public platform not linked to the health system, a resource such as InfoChagas could facilitate access not only for people with *T. cruzi* infection seeking for a second opinion or clarification of doubts not addressed within the constraints of daily health practice, but also for those who do not use the health services but are at risk of *T. cruzi* infection, who represent the majority of the users of the website.

We observed that website consultations peaked from 2019 to 2020, followed by a decline in the subsequent years. In contrast, YouTube channels rose after 2019. This trend likely reflects a growing reliance on digital platforms for healthcare information, following the onset of the pandemic, driven by factors such as increased internet accessibility, convenience, and efficiency [12,34,35]. In addition, telemedicine plays an important role in mobile populations, such as migrants, who represent a significant number of cases of *T. cruzi* infection [24,36]. [34,35]

These trends highlight the need to address digital health literacy gaps to improve access and outcomes among individuals infected with *T. cruzi*. A systematic review found that nearly 50% of participants across Latin America exhibit low health literacy, with similar challenges reported among Latin American migrants in the United States. In InfoChagas, the majority of users did not report *T. cruzi* infection, even in private consultations, suggesting that digital health literacy barriers may hinder full engagement with the platform, despite frequent mobile phone use [37,38].

The dual channel of consultation, website and YouTube channel, offered users different modes of interaction, with all queries across both platforms answered by trained professionals, ensuring information quality. The website predominantly received personal consultations, with 91.4% submitted on behalf of the users themselves. In contrast, the YouTube channel facilitated more impersonal consultations, and fostered community discussion and mutual support. The emergence of such virtual communities, is relevant for understanding the health process, as social representations constructed through communication with others shape how individuals construct reality [39].

### Barriers to healthcare access

The content analysis of the consultations provided insights into societal needs and demands, and through their narratives, revealed a strong limitation to provide a comprehensive system of care for *T. cruzi* infected individuals. Multidimensional barriers hinder the access to *T. cruzi* infection healthcare worldwide, perpetuating the low rates of diagnosis and treatment, and affecting the quality of care [36,40,41]. Systemic barriers accounted for the majority of the consultations in InfoChagas (93%), with fewer consultations related to structural, psychosocial, or clinical barriers, suggesting users perceive of the platform as a second professional opinion or an alternative system, where users seek solutions to systemic gaps rather than emotional or social support. Although InfoChagas could serve to guide and provide timely support through appropriate information, the limitations associated with the lack of prevention, healthcare, and long-term care strategies, as well as the low awareness of *T. cruzi* infection among health professionals, require a sustainable, policy integrated responses. Platforms like InfoChagas can help identify user-perceived needs and inform health systems to adapt interventions to strengthen neglected tropical disease care.

### Role of InfoChagas in IEC

Consultation analysis of the narratives identified an urgent need for IEC strategies, which were present in 97% of the consultations. Persistent myths associated with the infection based on outdated concepts remain prevalent in the population at risk. Despite the major shift from the identification of *T. cruzi* infection as a poverty-related disease and the exclusive treatment of acute cases, to the current One Health approach, urbanization phenomenon, a person-centered approach, and the prioritization of multidisciplinary strategies, the integral care has not been implemented in the plans of the national health systems of the most affected countries [6,7,32,36]. This lack of implementation has a major impact on the quality of care and perpetuates stigma and resignation aptitudes among both infected and at-risk individuals and health professionals. The development and implementation of appropriate IEC strategies should therefore be prioritized [6,7].

The WHO has recognized IEC as a key strategy in the control of *T. cruzi* infection and other NTDs, as a transversal component of all the interventions [1]. Contemporary IEC emphasizes participatory, empowering models over hierarchical, didactic approaches, facilitating behavior change and promoting individual agency in health decision-making [6,7,42]. Part of the success of virtual online platforms such as YouTube in health education lies in their incorporation of some of the elements described, democratizing access to a global audience, providing visual entertainment, and offering opportunities for interactive learning through comments and discussions, thereby increasing learner engagement [43]. It is therefore imperative that we transform our methods of communication by recognizing and adapting IEC to the diversity brought about by globalization and to the digital age. This would include considering linguistic, ethnic, cultural and territorial diversity, with the respect that different ways of living and understanding life, illness and death deserve.

## Limitations and opportunities for improvement

Although InfoChagas is a leading platform for IEC and patient support in *T. cruzi* infection, we identify several areas for improvement. First, the inclusion of additional languages beyond Spanish and English, such as Portuguese, Guarani, Quechua and or other local languages in Latin America would increase accessibility and align with the Sustainable Development Goals’ principle of leaving no one behind [44]. Second, the platform could increase its outreach to younger populations by incorporating participatory virtual content through social media [45,46]. Third, the inclusion of expert patients as respondents could strengthen the psychosocial support [47]. In addition, while the website has a systematic approach to responses, the YouTube channels are more unorganized, leading to missed or incorrectly answered queries by the public. The introduction of a trained artificial intelligence system could streamline common questions, reducing delays and improving coverage [48]. The introduction of a user evaluation system and periodical updates and user evaluation system could improve the service provided.

We acknowledge that our study design is primarily descriptive and cross-sectional, capturing a snapshot of the InfoChagas performance, influenced by the dynamic nature of YouTube and our limitation to obtain data from the website before 2019. The data period analyzed includes the COVID-19 pandemic, which may have led to an overuse of online services. In contrast, the use of digital health platforms is currently growth steadily, driven by two main social factors: trust and cost [49]. These factors are particularly relevant in *T. cruzi* infection, as they associated with the main barriers to accessing health services, which are difficult to overcome in the near future [36]. As the study relies on voluntarily submitted consultations, there may be some self-selection, making the tool not suitable as an epidemiological indicator. However, it demonstrates high potential underscoring insights into the populations affected. Lack of user demographic data, such as age and educational level, limited the analysis of across subgroups. Although the vector related consultation did not provide images, introducing a potential for misinterpretation, they were submitted within the information of InfoChagas, suggesting that this risk is minimal. Finally, we were not able analyze the resolution or timing of the responses, precluding evaluation of effectiveness.

## Conclusion

InfoChagas has served as a channel for information, education, and communication, and as a facilitator for community building among people affected by and at risk of *T. cruzi* infection in different geographical regions. The comprehensive analysis of consultations on the InfoChagas platform provides insights into the information and support needs expressed by users, revealing ongoing informational gaps and systemic barriers in accessing care. Addressing these needs through improved health system support, targeted educational initiatives and enhanced digital communication could strengthen public health interventions and IEC strategies to improve prevention, early diagnosis and the care provided. These findings underscore the potential role of digital platforms in supporting public health strategies, areas of research, informing policy decisions, and guiding resource allocation, while emphasizing the need for expert oversight and community engagement to maximize their effectiveness.
